# Height augmentation in 11β-hydroxylase deficiency congenital adrenal hyperplasia

**DOI:** 10.1186/s13633-015-0008-0

**Published:** 2015-05-15

**Authors:** Munier A Nour, Danièle Pacaud

**Affiliations:** Department of Pediatrics, College of Medicine, University of Saskatchewan, Saskatoon, Saskatchewan Canada; Division of Pediatric Endocrinology, Alberta Children’s Hospital, Calgary, Alberta Canada; Faculty of Medicine, University of Calgary, Calgary, Alberta Canada; Department of Pediatrics, College of Medicine, Royal University Hospital, 103 Hospital Drive, Saskatoon, SK S7N 0W8 Canada

**Keywords:** Congenital Adrenal Hyperplasia, Growth Hormone, Aromatase Inhibitor, Growth, Steroid 11-beta-Hydroxylase

## Abstract

**Context:**

11β-hydroxylase deficiency is the second most common form of congenital adrenal hyperplasia. Untreated, this enzyme deficiency leads to virilization, hypertension, and significant height impairment.

**Patient:**

We describe a patient from abroad who first presented to us at age 7 years for follow-up of ambiguous genitalia. He had been investigated and treated in Pakistan at 3-years-of-age following presentation for bilateral cryptorchidism. He was found to have 46, XX karyotype, elevated 17-OH progesterone and was diagnosed with congenital adrenal hyperplasia. In Pakistan, the patient had abdominal hysterectomy, bilateral salpingoophrectomy, and was started on corticosteroid replacement. At 7 years, shortly after immigrating to Canada, height was 138 cm and BMI 19.3 kg/m^2^ (+2.9 SDS and +1.7 SDS, respectively, male growth chart) and blood pressure was greater than the 99th percentile for age and height. The patient had Prader stage III - IV genital anatomy. Bone age was significantly advanced, yielding a severely compromised predicted final adult height. Biochemical evaluation was consistent with 11β-hydroxylase deficiency congenital adrenal hyperplasia.

**Intervention and outcome:**

In an attempt to improve final height, in addition to glucocorticoid replacement, this patient was treated with recombinant growth hormone and a third generation aromatase inhibitor (Letrozole) with an improvement in final height attained as compared with predicted height.

**Conclusions:**

This case of a 46,XX patient raised as male with congenital adrenal hyperplasia due to 11β-hydroxylase deficiency highlights a number of unique and difficult treatment challenges; specifically, the role of new therapeutic options for optimization of growth in the context of prior suboptimal disease management.

## Introduction

Congenital adrenal hyperplasia (CAH) due to 11β-hydroxylase (11β-OH) deficiency is the second most common cause of CAH [1]. It accounts for approximately 5-8% of all CAH cases and is caused by mutations in the *CYP11B1* gene [2]. The deficiency in 11β-OH results in a buildup of 11-deoxycortisol and 11-deoxycorticosterone. Clinical manifestations of CAH due to 11β-OH deficiency include virilization and ambiguous genitalia in females. The primary distinguishing feature between the more common 21-hydroxylase (21-OH) deficiency and 11β-OH deficiency is the presence of hypertension and hypokalemia in the latter.

Similar to CAH due to 21-OH deficiency, optimization of height in CAH due to 11β-OH deficiency remains a difficult balance between suppressing adrenal androgen production with corticosteroids and minimizing iatrogenic effects of supra-physiologic corticosteroid treatment. As a result, despite optimal treatment final adult height is often impaired [3].

We report a case of a male-identifying, 46, XX, 11β-OH deficiency CAH patient who presented to our tertiary care center recently after immigration. At the initial visit in our center, he had significantly compromised projected final adult height. In this report we highlight the significant response to height augmenting therapies, namely a combination of growth hormone (GH) and aromatase inhibitor (AI), with documented final adult height in our patient.

## Methods

Bone age assessment was independently assessed using the Greulich and Pyle methodology [4]. Predictions of final adult height were calculated use the Bayley and Pinneau method [5]. Laboratory assessments were completed through locally available laboratory services.

Patient care was provided through outpatient pediatric endocrinology clinic. All risks and benefits of interventions were discussed with patient and family. Appropriate consents were obtained for publication of this report.

## Case presentation

A 7-year-old male patient of Pakistani descent and consanguineous parents (first cousins) presented with ambiguous genitalia for evaluation to our endocrine clinic following assessment of a younger sister. The younger sister had initially been found to have ambiguous genitalia and hypertension while being evaluated in the emergency department for an intercurrent illness. The family had recently immigrated to Canada. The 7-year-old patient had initially received evaluation and treatment at 3-years-of-age while in Pakistan for bilateral cryptorchidism. At that time he was found to have normal electrolytes, a 46, XX karyotype and an elevated 17-OH progesterone (28 ng/mL; normal <1 ng/mL). He was diagnosed there with non-salt wasting CAH. The specific enzymatic deficiency was not delineated. The patient underwent an abdominal hysterectomy and bilateral salpingoophrectomy. He was started on corticosteroid replacement prior to moving to North America. According to his parents, follow-up was minimal and no corticosteroid dose adjustment was made between his initial presentation at 3 years and when he was first seen in Canada at 7-years-of-age.

**Figure 1:**
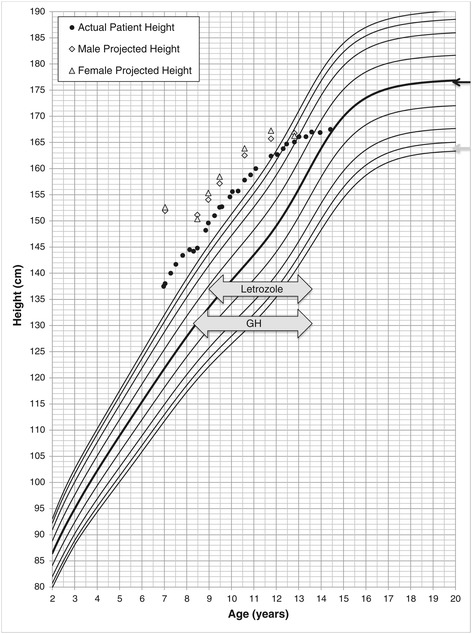
**Patient growth chart plotted on male growth chart depicting actual height and calculated predicted height using male (diamond) and female (triangle) reference standards** **[** 4 **,** 5 **].** Parental heights are depicted with grey (mother) and black (father) arrows on the right vertical axis. GH and Letrozole treatment duration depicted directly on the chart.

At 7 years, height was 137.5 cm (+2.9 S.D on male growth curve) (see Figure 1) and blood pressure was greater than the 99th percentile for age and height. The patient had Prader stage III - IV genital anatomy and Tanner stage 1 pubertal development. Baseline investigations demonstrated elevated 17-OHP, 11-deoxycorticosterone, 11-deoxycortisol, and suppressed plasma renin activity (See Table 1). There was significant bone age advancement to 12 years (female standard [4]; see Table 2), for chronological age of 7 years, yielding a predicted final adult height of 152.6 cm (−3.3 S.D. for a male) [4]. Findings were consistent with 11β-OH deficiency CAH.

**Table 1:** **Laboratory investigations results at time of presentation at 7 years of age**

**Test**	**Value**	**Reference range**
Androstenedione (nM)	7.7	<2.0
Testosterone (nM)	2.2	<1.0
17-OH Progesterone (nM)	30.6	<6.0
11-Deoxycortisol (nM)	1580	<4.5
11-Deoxycorticosterone (nM)	40.3	<1.03
Plasma Renin Activity (ng/L/s)	0.15	>0.28

**Table 2:** **Comparisons of final height estimations by male and female interpretations of bone age radiographs** [4,5]

**Chronological age (years)**	**Height (cm)**	**Female bone age (years)**	**Estimated final height (cm)**	**Male bone age (years)**	**Estimated final height (cm)**
7	137.5	12	152.6	14	151.9
8.5	144.8	13.5	150.4	15	151.1
9	149.6	13.5	155.3	15.5	154.1
9.5	152.6	13.5	158.5	15.5	157.2
10.6	157.8	13.5	163.9	15.5	162.5
11.8	162.4	14	167.1	16	165.7
12.8	165.1	16	166.3	17	166.8

### Intervention & results

The patient was treated with glucocorticoids while in Pakistan, however, treatment was suboptimal. On initial visit in Canada corticosteroid replacement was optimized to a replacement dose of approximately 15 mg/m^2^/day of hydrocortisone. Doses were adjusted as necessary and increased to approximately 18–20 mg/m^2^/day of hydrocortisone on average. This was subsequently changed to ~3.5 to 4.1 mg/m^2^/day of prednisone (using a bioequivalence ratio of 5, this equates to 17.5 to 20.5 mg/m^2^/day of hydrocortisone). Despite this treatment, ongoing bone-age advancement and a slowing growth velocity to 4.2 cm/year resulted in further diminishment of the projected adult height to 150.4 cm (−3.7 S.D.).

After lengthy multidisciplinary discussions with the family surrounding the ethical, social and ethnic considerations of growth optimization, the decision to attempt growth augmentation was undertaken. GH treatment (0.05 mg/kg/day; 6 days per week) was initiated at 8.3 years, which resulted in an increase in growth velocity to 7.8 cm/year. However, in response to continued bone age advancement, an AI, Letrozole (2.5 mg daily), was initiated at 9 years of age.

Blood pressure remained elevated despite glucocorticoid replacement and further treatment with Spironolactone and Amlodipine were required for control. At age 12 the patient had insertion of testicular prosthesis. At age 14 hypospadias repair and release of chordae was performed. In addition, removal of breast tissue was performed at this time as well. The patient was not initiated on testosterone supplementation until completion of growth as endogenous androgen production was deemed to be adequate and to help maximize growth potential. Of note, the circulating adrenal androgens were enough to induce progressive pubertal changes such as axillary, pubic and facial hair growth, voice changes and acne.

The patient had no adverse effects from GH or AI treatment including no evidence of dysglycemia, dyslipidemia, hepatic dysfunction, vertebral malformations, nor evidence of bone fragility. Letrozole and GH treatment were continued until growth velocity was negligible at 13.6 years of age. A final adult height of 167.5 cm (−1.3 S.D. male chart) was achieved (See Figure 1). This represented an increase of 17.1 cm (2.6 S.D.) when using female bone age standards or 16.4 cm (2.3 S.D.) when using male bone age standards from the projected final adult height before introducing GH and AI treatment (Table 2).

## Discussion

A number of complex treatment issues arise in the management of patients with CAH. Issues such as gender-identity, growth, blood pressure management, surgical correction, and pubertal management are among the clinical difficulties addressed by the multidisciplinary medical team. The presented case illustrates a number of these in a young patient with CAH due to 11β-OH deficiency. Among these issues, in particular, we highlight the unique aspects of optimizing height in this patient with dramatic improvement in final adult height.

The influence of CAH due to 11β-OH deficiency on final adult height has not been thoroughly examined in the literature. While no reviews exist in 11β-OH deficiency, a recent systematic review and meta-analysis of 21-OH deficiency CAH demonstrated the final height S.D. score was – 1.38 (−1.56 to −1.20; I^2^ = 90.2%) in patients, despite optimization of CAH treatment [6]. Optimization of height is often a delicate balance between corticosteroid replacement to suppress androgen excess while attempting to limit effects of glucocorticoid excess. The treatment of 11β-OH deficiency involves achieving a similarly challenging balance. Although, as demonstrated with the presented patient, CAH due to 11β-OH deficiency may require higher glucocorticoid doses than those currently recommended for patients with CAH due to 21-OH deficiency [7]. Patients with suboptimal control or inadequate treatment, such as our patient, may have substantial reduction in their growth potential [3].

Current Endocrine Society guidelines do not endorse the use of growth augmenting therapies outside of the context of research studies [7]. Our case highlights the success of these treatments in the setting of late diagnosis and initial suboptimal control leading to a significant threat to final height attainment. While we do not advocate for such therapies in all CAH patients, in certain dire circumstances their use may be justified. Prior to initiation of GH and Letrozole, our patient had a predicted adult height of 150.4 – 151.1 cm (depending on female or male reference standard used respectively) which increased by 16.4 – 17.1 cm by the time of attainment of final adult height (167.5 cm; −1.3 S.D. male standard). This final height is considerably greater than these early predictions and also higher than his genetic calculated mid-parental height for a female of 164 cm (−1.8 S.D.). He received a total of 5.3 years of GH treatment and 4.6 years of AI treatment. A recent prospective study of GH use in 21-OH deficiency CAH found a final height gain of 9.2 ± 6.7 cm in males and 10.5 ± 3.7 cm in females with a mean GH treatment duration of 5.6 ± 1.8 and 4.5 ± 1.6 years, respectively [8]. We would advocate that in the setting of extreme impairment of predicted final height, consideration of these therapies may be beneficial. Similar to dosages used in idiopathic short stature, we used a relatively high dose of GH in our patient given the degree of predicted growth impairment.

Our patient benefited from a combined therapeutic approach using corticosteroids, GH and aromatase inhibition. Growth hormone treatment was initiated due to ongoing impairments in predicted final adult height despite corticosteroid treatment. While the GH treatment had benefit in growth velocity (4.2 cm/year vs. 7.8 cm/year respectively) further advancement in bone age continued to compromise projected height benefit. The addition of Letrozole, a third generation steroidal AI, to the treatment regime resulted in dramatic benefit in final height by inducing a slowing bone maturation. Over the span of approximately 2 years there was virtually no advancement of bone maturation.

Aromatase inhibition has been used in an off-label manner in a select group of pediatric conditions, including hyperestrogenism, hyperandrogenism, pubertal gynecomastia and short stature [9]. Their use in CAH has been limited. To date only one study has been conducted examining the use of AIs for height augmentation in CAH [10]. In this trial, children were randomized to 2 year treatment with 1) Flutamide, testolactone and reduced hydrocortisone dose and fludrocortisone vs. 2) Standard treatment with hydrocortisone and fludrocortisone. The study demonstrated a slight trend to increased predicted final adult height (+0.6 S.D.) in the testolactone/flutamide group, however, the difference was not statistically different. This trial has been ongoing and in 2004, subjects were switched to Letrozole; however, results have not yet been published [11].

The use of AIs in the pediatric setting must be used with caution due to unknown potential long term complications and thus far only modest height benefits. In males treated with AIs, hypertestosteronemia has been observed [12]. This rise in serum testosterone is due to the reduced estrogen feedback on the hypothalamus leading to increased levels of LH. No adverse effects from hypertestosteronemia have been reported. In our patient, previous oophorectomy and female karyotype obviated this risk. Of interest, however, a slight increase in serum androgens was noted with AI introduction. This increase would be possible either through decreased conversion of androgens or an increase in adrenal androgen production. Other potential concerns with AI use have included impaired bone mineral density, body composition and metabolic effects such as dyslipidemias [9]. In addition, there is hesitancy in using AIs in females due to concerns of undesirable effects of hyperandrogenemia. In our patient, these potential effects were viewed as desirable due to his male gender identification. Vertebral malformations have also been reported to be associated with the use of AIs with particular risk when used during the pre-puberty or early puberty phases [13].

Various other growth augmenting therapies have been explored in the setting of CAH with variable success. In addition to standard therapy with glucocorticoids, these have included growth hormone, GnRH analogs, and anti-androgens to increase final height [14]. In our patient, GnRH analogs would have been of no benefit given the previous oophorectomy.

Of particular concern for our patient were the cultural and societal implications of height attainment. Being raised male, our patient had societal expectations of a higher final height attainment. While evolutionarily this increased final height in males is less drastic than in other species, it remained an important factor for this family when determining treatment goals [15]. The notion that short stature has a negative impact on psychosocial adaptation has been disproved by recent studies done in healthy children [16]. However, in the context of short stature associated with a chronic condition such as CAH, patient and family preferences may need to be considered. In particular, a patient already stigmatized with having a chronic medical condition, gynecomastia, infertility, and abnormal genital anatomy, the normalization of height was an attainable and beneficial goal for patient care.

While assessing bone maturation remains an important tool in monitoring treatment in CAH [17], considerable difficulty exists in determining the most accurate method to predict final adult height, particularly in patients such as the one presented. Although our patient was raised as male, he had a 46, XX karyotype. The endogenous androgen exposure of our patient was significantly higher than that of an otherwise healthy female. These exposures may have significant influence on bone growth and development. Thus, the interpretation of bone age radiography may be difficult as the differential effect of chromosomal and hormonal influences are ultimately unknown. Interestingly, despite significant variations in bone age interpretation based on gender, the effect on final height prediction for our patient was minimal (see Table 2 and Figure 1). Ultimately, consistency with regard to interpretation of these tests is paramount.

## Conclusions

In summary, we present the growth response of a 46, XX patient with CAH due to 11β-OH deficiency to final adult height. Despite the significant impairment in predicted adult height due to inadequate initial treatment, this patient demonstrated favorable results with a combined therapeutic approach including the addition of GH and an AI to the standard glucocorticoid treatment to optimized growth. Our patient’s response to this regimen underscores the need for further research into these new therapeutic avenues.
